# Expression and clinical significance of c-Met in advanced esophageal squamous cell carcinoma

**DOI:** 10.1186/s12885-014-1001-3

**Published:** 2015-01-15

**Authors:** Yingying Xu, Zhi Peng, Zhongwu Li, Ming Lu, Jing Gao, Yilin Li, Yanyan Li, Lin Shen

**Affiliations:** Department of Gastrointestinal Oncology, Key Laboratory of Carcinogenesis and Translational Research (Ministry of Education), Peking University Cancer Hospital & Institute, FuCheng Road 52, HaiDian District Beijing, China; Department of Pathology, Key Laboratory of Carcinogenesis and Translational Research (Ministry of Education), Peking University Cancer Hospital & Institute, Beijing, China

**Keywords:** Esophageal squamous cell carcinoma, c-Met, Chemotherapy, Overall survival

## Abstract

**Background:**

c-Met, one of current potential hot targets, has been suggested as a potential tumor marker in the development of esophageal squamous cell carcinoma (ESCC). Our aim was to investigate the expression of c-Met in advanced esophageal squamous cell carcinoma in four phase II trials who had tumor tissues from archival in our center and analyze the correlations between c-Met expression and clinical features.

**Methods:**

Ninety patients with advanced ESCC who were admitted to the phase II clinical trials in the Department of Gastrointestinal Oncology, Peking University Cancer Hospital and Institute from March 2007 to March 2014 were finally eligible for present study and the corresponding tissues and clinical data were collected. The expression of c-Met in the tissue samples was detected by immunohistochemistry (IHC). c-Met overexpression was defined as ≥ the median value of H-score. Kaplan-Meier and Cox multivariate regression were conducted to evaluate the relationship between c-Met expression and ESCC survival.

**Results:**

The overexpression of c-Met is 43.3% in advanced ESCC. There was no statistical difference between c-Met expression and clinical features except sex and tumor location. Survival analysis documented that the overexpression of c-Met predicted a worse prognosis (OS: 253 d *vs* 422 d, *P* = 0.011). In the group treated with chemotherapy combined with anti-EGFR drugs, patients with lowexpression of c-Met had a better OS than those with overexpression of c-Met (OS: 577 d *vs* 232 d, *P* = 0.007).

**Conclusions:**

c-Met may be an independent prognostic factor in advanced ESCC. The overexpression of c-Met may predict a worse efficacy of anti-EGFR therapy.

## Background

Esophageal carcinoma is one of the most common gastrointestinal cancers, the incidence of which ranks the fifth among all of the malignant cancers in China [1]. More than 95% of esophageal cancer in China is esophageal squamous cell carcinoma (ESCC). Due to the atypical clinical symptoms, most patients develop into advanced stages when first diagnosed and lost operation opportunity. Systematic therapies, including chemotherapy, are of great importance for patients with advanced ESCC, whereas the efficacy of chemotherapy based on 5-FU/DDP/PTX is limited. As the ubiquity poor prognosis of advanced ESCC, new strategies are urgently needed, especially of targeted-therapy. c-Met is one of the most important factors with cancer-associated receptors and pathways. c-Met tyrosine kinase is the cell-surface receptor for hepatocyte growth factor (HGF) which is involved in regulating cell proliferation, apoptosis, and migration. c-Met activity is normally detected in defined stages of embryogenesis and organogenesis [2-7]. HGF and c-Met have a significant relevance to lymph node stage and distant metastasis. It was reported that c-Met was involved in a number of human primary tumors, including gastric, breast, colorectal, liver and renal cancer [8].

Recently, a meta-analysis focusing on c-Met overexpression and the prognosis of gastric cancer including 14 studies [9] indicated that higher amplification and expression of c-Met gene in gastric cancer is an indicator of poor prognosis. c-Met was overexpressed in 34%-54% of esophageal adenocarcinoma and had a significant association with survive [10]. c-Met overexpression in ESCC is about 7% and not correlated with prognosis in western countries. Our study in China showed that c-Met overexpression in ESCC is about 34% [11], which differs from western countries. However, by now, there is no study on the c-Met expression and clinical significance in advanced ESCC.

Therefore, in this study, we detected c-Met expression in advanced ESCC patients who involved in the phase II clinical trials in our center. We investigated the expression levels of c-Met in advanced ESCC and estimated the relationship between c-Met expression and clinical features.

## Methods

### Patients and tumor specimens

Tumor samples were retrospectively identified from patients with ESCC who were enrolled in four phase II clinical trials between 2007 and 2014 at the Peking University Cancer Hospital in Beijing, China. Four phase II clinical trials for first-line treatment of ESCC were involved: Paclitaxel and Cisplatin in Patients With Advanced Squamous-Cell Carcinoma of the Esophagus [12]; Irinotecan Combined With Cisplatin as 1st Line Treatment for Esophageal Squamous Cell Cancer: a Single Center Prospective Clinical Trial (NCT01051765); Study of Nimotuzumab to Treat Esophageal Squamous Cell Carcinoma (NCT01993784); Sequential Paclitaxel Chemotherapy and Radiotherapy as 1st Line Treatment for Elderly Esophageal Squamous Cell Cancer (NCT02016287). The inclusion and exclusion criteria were as follows:

Inclusion criterion:

1. Histological confirmed stage IV according to AJCC 6.0 or recurrent ESCC.
2. Patients have not received any palliative chemotherapy or radiotherapy before.
3. Adjuvant therapies should be received more than 6 months before recurrence.

Exclusion criterion:

1. Adjuvant therapies are received within 6 months before recurrence.
2. Patients who have other primary carcinomas except ESCC.
3. Histology confirmed mixed tumor.

Anti-total c-Met (SP44) rabbit monoclonal primary antibodies were purchased from Ventana Company (Ventana Medical System, Tucson, USA).

Tissue samples for diagnostic purposes were obtained with the consent of each patient. All tumor specimens were fixed in 10% buffered formalin, embedded in paraffin and then made into continuous 4 μm tissue sections for IHC examination.

All patients signed written informed consent for their information to be used for study. Study approval was obtained from independent ethics committees at Peking University Cancer Hospital. The study was undertaken in accordance with the ethical standards of the World Medical Association Declaration of Helsinki.

### Immunohistochemistry (IHC)

IHC evaluation was performed using anti-total c-Met(SP44) rabbit monoclonal primary antibody. The staining was carried out according to the manufactures’ protocol on the BenchMark XT platform from Ventana utilizing the ultraView detection kit. The c-Met staining intensities were evaluated by two pathologists who were blinded to the diagnosis of individual patients. c-Met was localized primarily in the cytoplasm and membrane. Specifically, intensity was scored according to a four-tier systems: 0, no staining; 1+, weak; 2+, moderate; and 3+, strong.

To our knowledge, there were no validated scoring systems for interpretation of c-Met staining intensity. Here, positive results were judged by two systems of criterion.

1. H-score assessment was based on staining intensity(0–3) and the percentage of positive cells (0-100%). Each individual intensity level was multiplied by the percentage of cells and all values were added to obtain the final IHC score, ranging from 0 to 300. The final score was calculated from the scores of assessment at membranous and cytoplasmic expression.
2. The other evaluation of c-Met was performed according to MetMab IHC defined scoring criteria (positively defined as having ≥ 50% of tumor cells positive for membranous or cytoplasmic and/or c-Met immunostaining with moderate or strong intensity, i.e. ≥ 2+) in the clinical trial of NCT01456325, a MetMab phase III trial in advanced NSCLC [13].

### Statistical analysis

A number of prognostic factors between different c-Met expression groups were examined in univariate analysis. These factors included: (1) sex: male and female; (2) baseline age, dichotomized as ≤59 years vs. >59 years; (3) tumor location; (4) presence of distant metastasis; and (5) presence of lymph node invasion. Comparison of categorical variables was performed with the chi-square test or the Fisher’s exact test. Initial analysis involved a univariate investigation of relationship between c-Met overexpression and OS time using Kaplan-Meier survival curves. To identify independent biomarkers, multivariate analyses adjusted for the baseline variables were performed using a Cox regression model for OS. *P* values of <0.05 were considered significant. All analysis were performed using SPSS 19.0 software (IBM, Armonk, NY).

## Results

### Patients’ characteristics and c-Met expression

**Figure 1:**
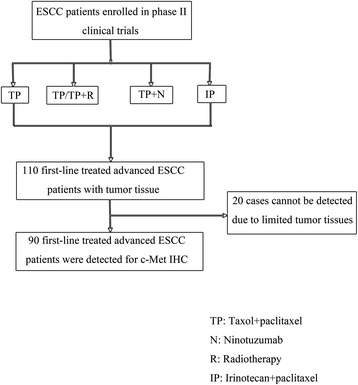
**The patients screening process.**

**Figure 2:**
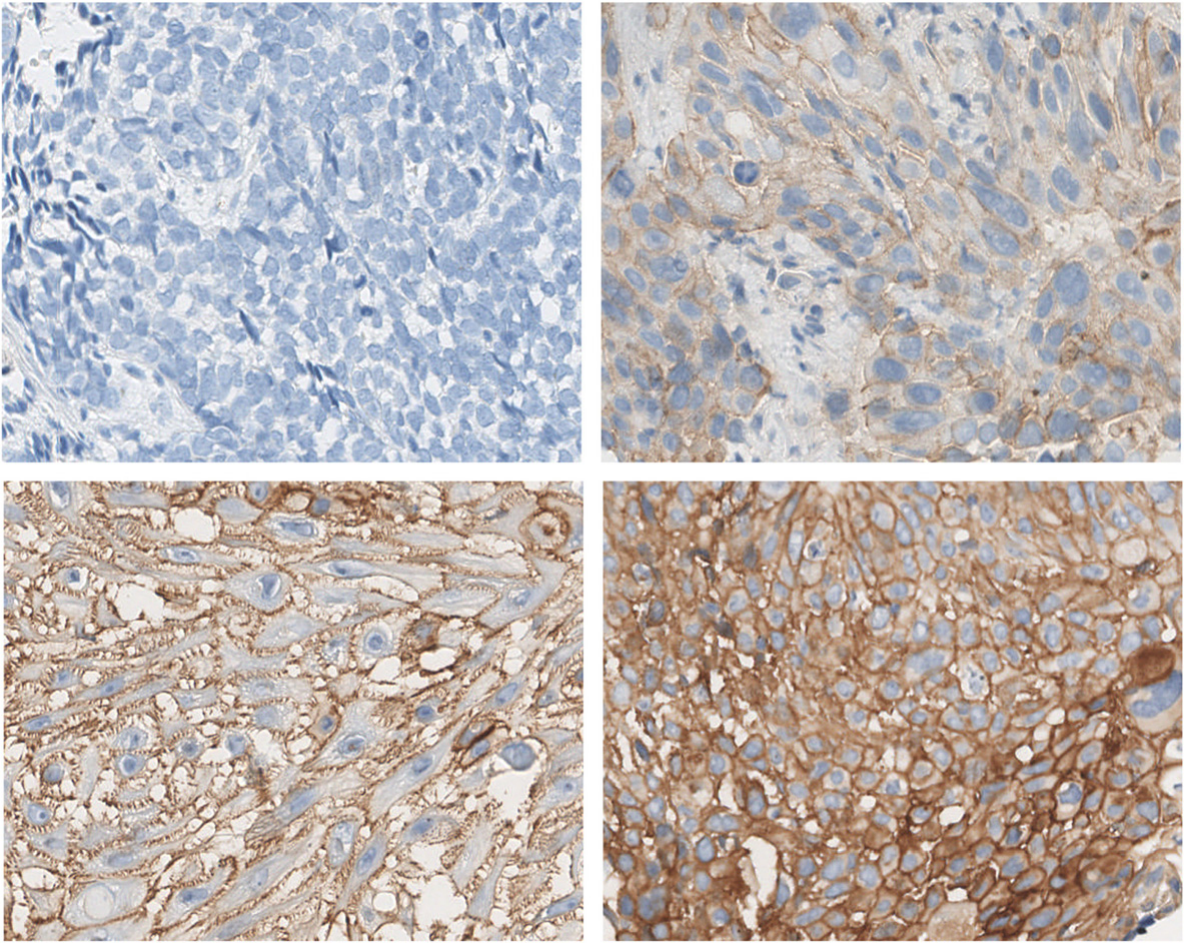
**The representative c-Met staining intensities were localized primarily in the cytoplasm and membrane.** Intensity: 0, no staining; 1+, weak; 2+, moderate; and 3+, strong.

After screening all the patients’ medical record, one hundred and ten cases were detected for c-Met IHC (Figure 1). Due to limited tumor tissues, ninety cases were successfully detected. The representative IHC intensity of c-Met expression is described in Figure 2. All patients were followed up till Nov 6, 2014. Of the 90 patients, 63 died, 19 survived, and 8 were lost.

H-score of c-Met IHC ranged from 0 to 270, with the median value of 20, which was chosen as the cutoff point for separating c-Met over-expression tumors from c-Met low-expression tumor. Of the 90 patients, 51 cases had an H-score ≤ 20, considered as IHC low-expression, and 39 cases had an H-score > 20, considered as IHC over-expression. No statistically significant difference of c-Met expression was found between different groups of sex, age, tumor location, tumor differentiation, lymph node invasion and distant metastasis (Table 1).

**Table 1:** **The relationship between c-Met expression and clinical features of ESCC (according to H-score)**

	**N**	**H-score ≤ 20**	**H-score > 20**	***P***
Sex				
Male	73	39	34	0.198
Female	17	12	5	
Age				
≤59	45	25	20	0.832
>59	45	26	19	
Tumor location				
Upper thoracic	6	4	2	0.489
Middle thoracic	48	24	24	
Lower thoracic	35	22	13	
Abdominal	1	1	0	
Tumor differentiation				
Poor	31	16	15	0.769
Moderate	46	27	19	
Well	13	8	5	
Distant metastasis				
Yes	27	13	14	0.286
Liver	14	8	6	0.969
Lung	19	8	11	0.114
No	64	38	25	
Lymph node invasion				
Yes	85	48	37	0.877
No	5	3	2	

### c-Met expression correlated with patient survival in advanced ESCC

The relationship between OS and clinical features of ESCC patients was analyzed through log-rank and chi-square test. The results showed that no statistical significance of OS was found in different groups of age, stage, distant metastasis, tumor differentiation. However, OS was significantly different by sex and tumor location. Female patients had a better prognosis than male patients (577d *vs* 333d, *P* = 0.041). Patients with tumor located in upper thoracic (475d) and middle thoracic (422d) had a better prognosis than those in lower thoracic (258d) (*P* = 0.005).

**Figure 3:**
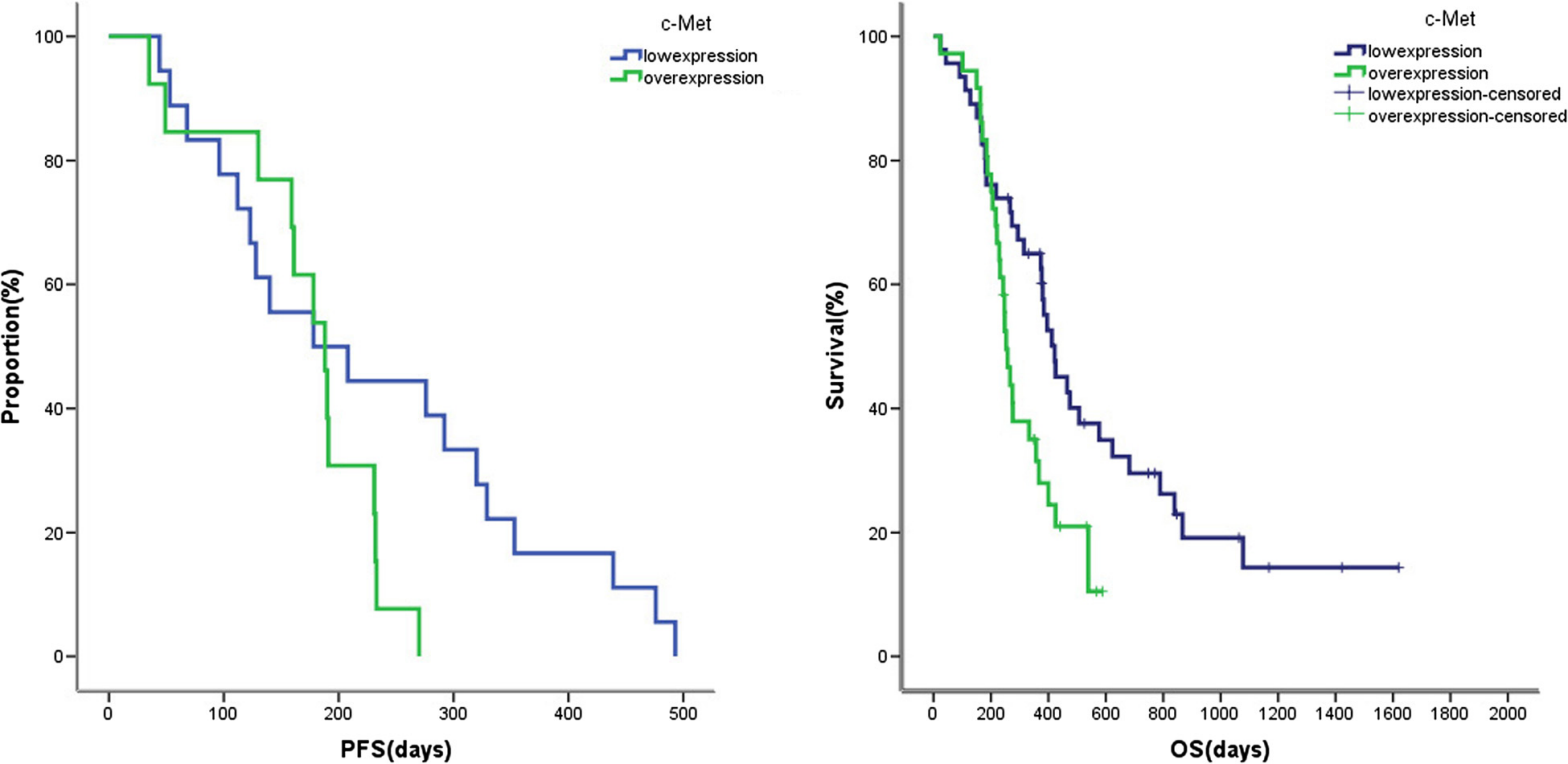
**Kaplan-Meier survival curves of patients with ESCC according to c-Met expression.**

Progress-free survival(PFS) was not significantly different between the two c-Met expression groups (the median PFS of c-Met over-expression *vs* c-Met low-expression: 188d *vs* 178d, *P* = 0.089). The median OS was 333d (95% CI 252d-482d). The OS was closely related to the expression of c-Met in ESCC tissues. As shown in Figure 3, the survival rate of c-Met over-expression patients was significantly lower than that in the low-expression group (253d *vs* 422d, *P* = 0.011). In the multivariate Cox regression model adjusted for baseline variants, there was still a statistical significance between OS and c-Met expression (HR = 1.805, 95% CI = 1.045-3.117, *P* = 0.034).

**Figure 4:**
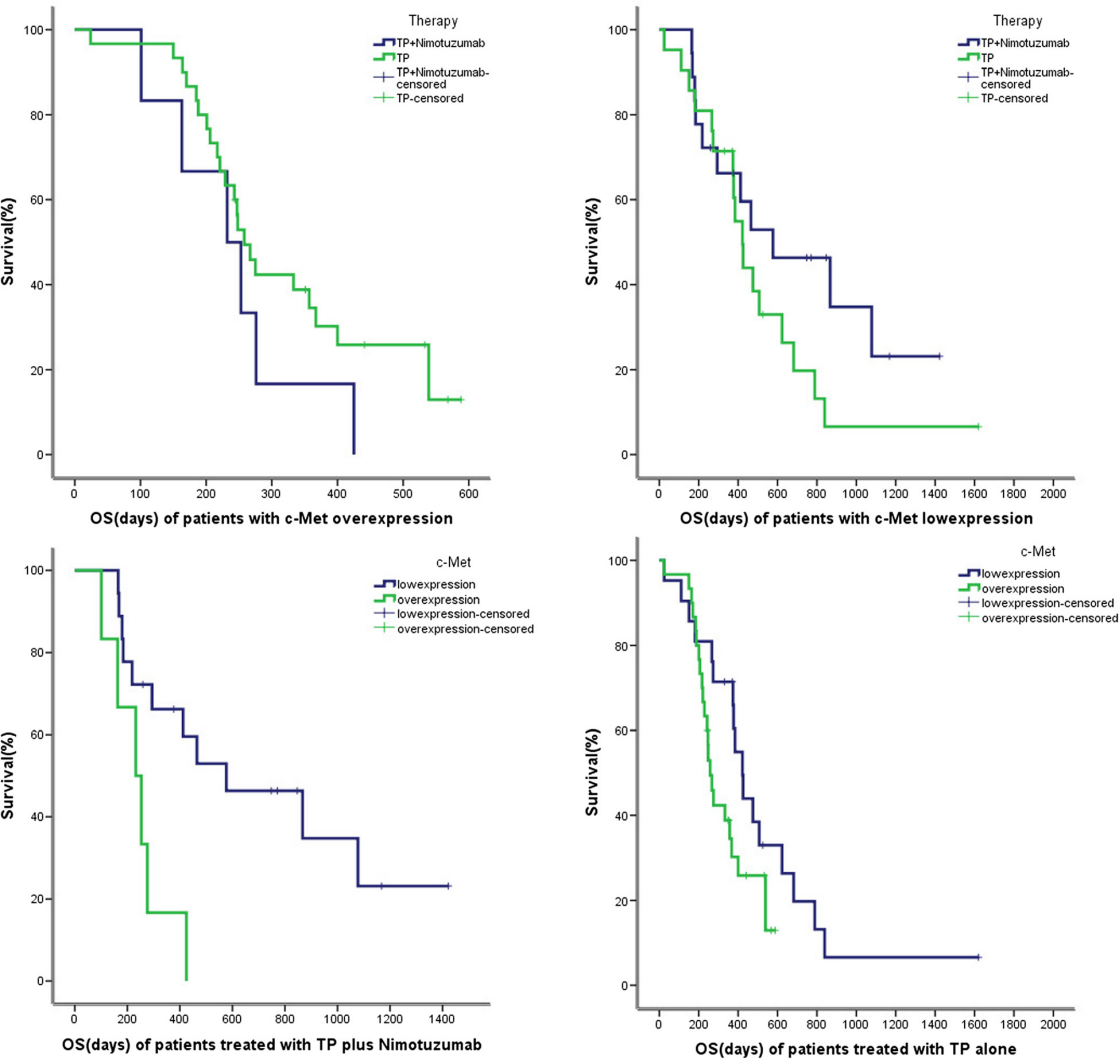
**Kaplan-Meier survival curves of patients with ESCC according to treatment and c-Met expression.**

In this study, 25 patients received chemotherapy of taxol and cisplatin (TP) plus nimotuzumab, 55 patients received chemotherapy of TP alone, and 10 patients received other chemotherapies. In c-Met overexpression group, OS of patients who received TP plus nimotuzumab was not different from that of patients who received TP alone (232d *vs* 258d, *P* = 0.221). Similar results were obtained in c-Met low-expression group (577d *vs* 422d*, P* = 0.152). In TP plus nimotuzumab group, the OS of patients with c-Met low-expression was significantly better than those with c-Met over-expression (577d *vs* 232d, *P* = 0.007). In TP alone group, no statistical significance was found (422d *vs* 258d, *P* = 0.076) (Figure 4).

The other evaluation of c-Met was performed according to MetMab IHC defined scoring criterion. According to this criterion, 16 patients (17.8%) had c-Met over-expression and 74 patients (82.2%) had c-Met low-expression. There was no significant relevance between c-Met expression and different groups of age, sex, tumor location, tumor differentiation, distant metastasis. The patients with c-Met low-expression had a trend of better prognosis with no statistical significance (*P* = 0.289).

## Discussion

c-Met expression has been reported in a number of human primary tumors, including gastric, breast, colorectal, liver and renal cancer. c-Met plays an important role in tumor development and metastasis [8]. As the only receptor of HGF, c-Met kinase activation resulting in activation of downstream signaling which intermediates such as mitogen-activated protein kinase (MAPK), mammalian target of rapamycin (mTOR) pathway, and signal transducer and activator of transcription (STAT) pathway which leads to changes in gene expression and cell behavior, like increased proliferation, survival, motility, invasiveness, and stimulation of angiogenesis.

Our study detected c-Met expression in 90 ESCC patients and analyzed the relationship between c-Met expression and clinical features and prognosis. According to H-score assessment, 43.3% of patients had c-Met overexpression, similar to other reports in China [11]. The most important finding in this study was that c-Met overexpression was associated with shorter OS in the patients with advanced ESCC, which may give clues for target therapy in advanced ESCC.

One study reported that an increased expression of c-Met was seen along the metaplasia-adenocarcinoma sequence and patients with esophageal adenocarcinoma with c-Met positive tumors showed lower 6-month survival rates after surgical resection than those with c-Met negative tumors [14]. There were few reports on ESCC in western countries. Mesteri reported that 7.6% of ESCC patients had c-Met over-expression, but c-Met plays no relevant role in ESCC [10]. However, there are no metastatic patients included in this study and the difference between stages may cause the different results. In China, more than 95% esophageal cancer patients are ESCC. c-Met overexpression of ESCC in China ranges from 34% to 58%. Our study had similar results. Dong G finds [15] that metastatic SCC cells that overexpress c-Met exhibit angiogenesis factor expression and enhance scattering in response to HGF in vitro, and tumorigenesis and metastasis in response to HGF in the tumor microenvironment in vivo. Peng [9] did a meta-analysis on c-Met expression in gastric carcinoma and found that higher c-Met gene amplification and expression in gastric cancer was an indicator of poor prognosis. Up to now, there is no other study focused on the relationship between c-Met expression and prognosis in ESCC in China. Further research still needs.

In our study, patients with c-Met lowexpression had a better OS than those with c-Met overexpression both in TP plus nimotuzumab group and in TP group. In c-Met overexpression group, patients treated with TP plus nimotuzumab had a worse OS than those treated with TP alone. On the contrary, in c-Met lowexpression group, patients treated with TP plus nimotuzumab had a better OS than those treated with TP alone. This suggests that patients with c-Met overexpression may not be suitable for anti-EGFR treatment. There has been no study on anti-EGFR treatment and c-Met expression before. However, a study finds out that in lung cancer, amplification of c-Met causes gefitinib resistance by driving ERBB3-dependent activation of PI3K, a pathway thought to be specific to EGFR/ERBB family receptors [16]. Recently, another study in colorectal cancer highlights the role of c-Met in mediating primary and secondary resistance to anti-EGFR therapies and encourages the use of c-Met inhibitors in patients displaying resistance as a result of c-Met amplification [17]. In the breast cancer, c-Met contributes to trastuzumab resistance, as inhibition of c-Met sensitizes cells to trastuzumab-mediated growth inhibition [18]. High gene copy numbers of c-Met and HGF associate with an increased risk of trastuzumab-based therapy failure in HER2-positive metastatic breast cancer [19]. In gastric cancer, HGF activation of c-Met receptors rescues cells from lapatinib-induced growth inhibition by restimulating the downstream pathways and restoring normal cell-cycle progression. This rescue effect could be abrogated by inhibiting c-Met with PHA-665752 (a highly specific c-Met inhibitor) or down-regulating c-Met expression with siRNA [20]. These mechanisms above may exist in ESCC which cause anti-EGFR drugs resistance. Further confirmation is needed.

There were some limitations in this study. First, this study was conducted in a relatively small sample size. Secondly, due to a retrospective study, only cancer tissues before treatment were detected. It would be better that cancer tissues after treatment, lymph nodes, distant metastasis, and normal tissues adjacent to cancer could be detected for c-Met IHC and c-Met gene amplification. On the other hand, information of patients treated with different therapies was not well matched. Thus, our findings should be validated in subsequent prospective studies in future before clinical application.

In short, c-Met plays an important role in development of ESCC and may have an effect on anti-EGFR therapy, which needs further studies.

## Conclusion

Patients with c-Met over-expression had a poorer prognosis than those with c-Met low-expression, suggesting that c-Met may be an independent prognostic biomarker in ESCC. Patients with c-Met over-expression treated with nimotuzumab had a shorter OS, indicating c-Met might be a predictor of efficacy of anti-EGFR therapy.
